# Supplementary material for: Single-particle tracking reveals heterogeneous PIEZO1 diffusion
Source: Biophys J. 2025 Jan 21;124(24):4457–70. doi: 10.1016/j.bpj.2025.01.010 (PMC12820997; doi:10.1016/j.bpj.2025.01.010)
Supplement: Document S1. Figures S1–S5 and Tables S1 and S2 [file mmc1.pdf]

Biophysical Journal, Volume *124*

Supplemental information

Single-particle tracking reveals heterogeneous PIEZO1 diffusion

Alan T. Ly, J. Alfredo Freites, Gabriella A. Bertaccini, Elizabeth L. Evans, George D. Dickinson, Douglas J. Tobias, and Medha M. Pathak

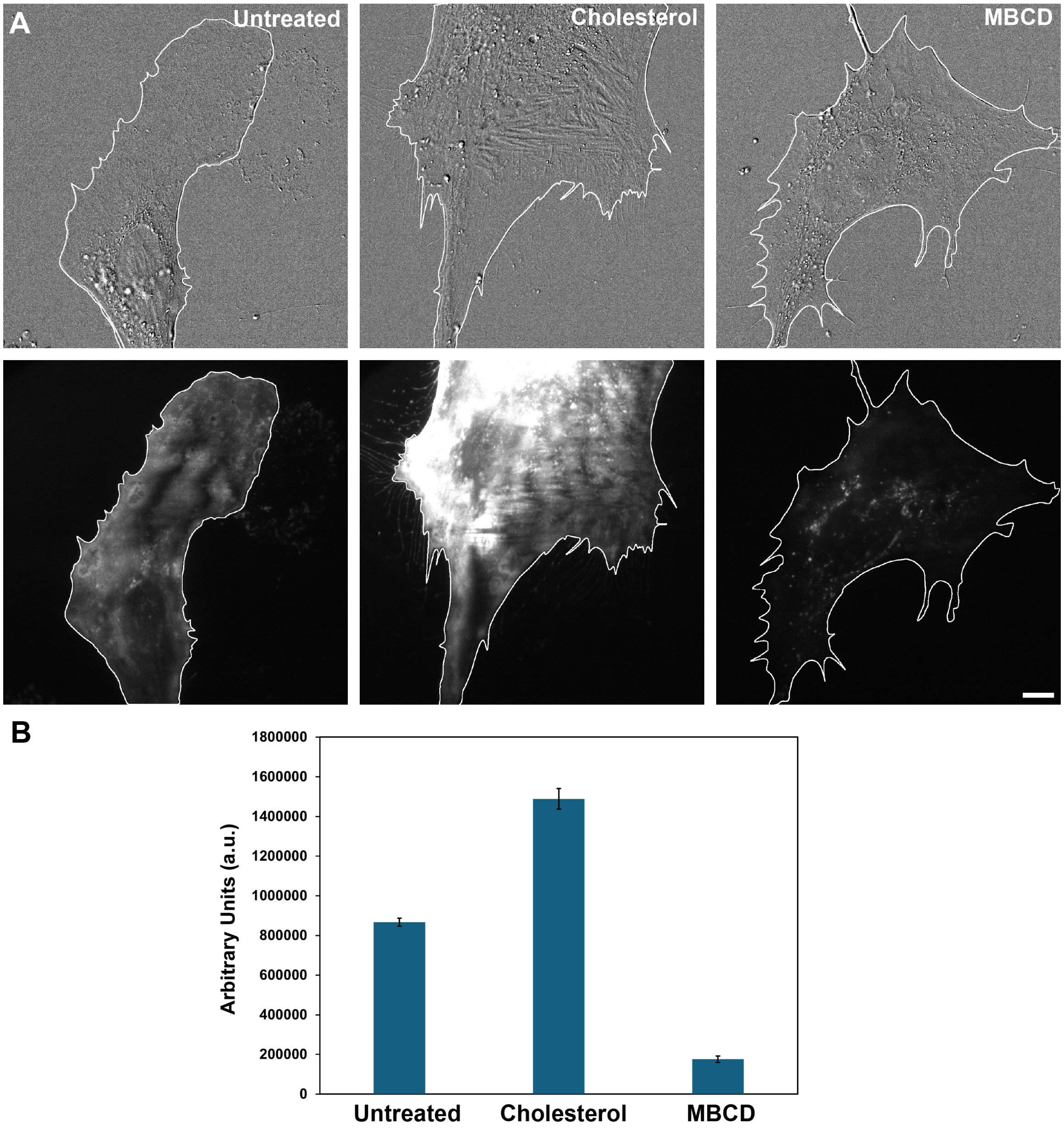

Supplemental Figure 1: Cholesterol supplementation via cholesterol incubation and cholesterol reduction via MBCD increases and decreases cholesterol signal respectively as determined using Filipin III. A. Representative DIC and TIRF microscopy images of PIEZO1-tdTomato MEFs stained for free cholesterol using Filipin III in the untreated, cholesterol-treated, and MBCD-treated conditions respectively. Cell boundary is denoted by white line. TIRFM images were brightness contrast adjusted the untreated condition. Scale bar = 10 µm. B. Mean calculated total cell fluorescence (CTCF) of Filipin III fluorescence. Intensity calculations were performed using Fiji (ImageJ). Untreated control (n=15), cholesterol-treated (n=16), MBCD-treated (n=15). Units are in arbitrary units (a.u.). Error bars represent mean CTCF ± SEM.

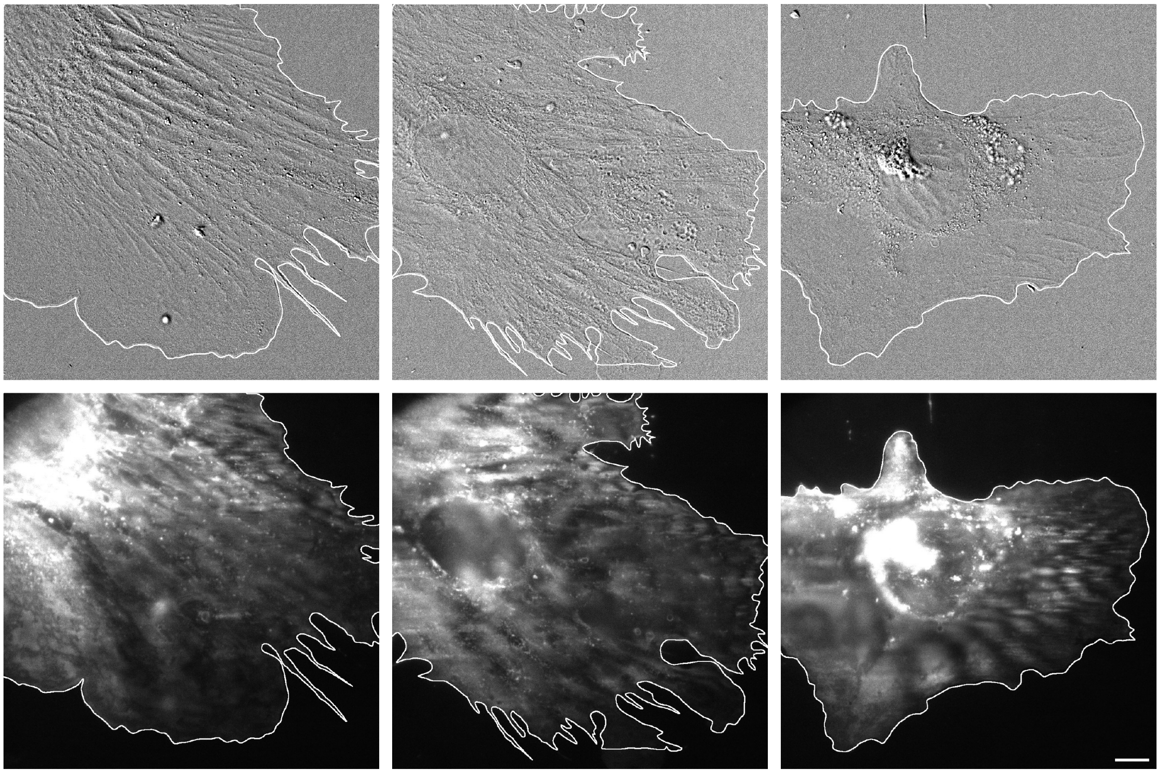

Supplemental Figure 2: Representative DIC and TIRF microscopy images of PIEZO1-tdTomato MEFs stained for free cholesterol with Filipin III in the cholesterol-treated condition. Cell boundary is denoted by white line. TIRFM images were brightness contrast adjusted to the untreated condition. Scale bar = 10 µm.

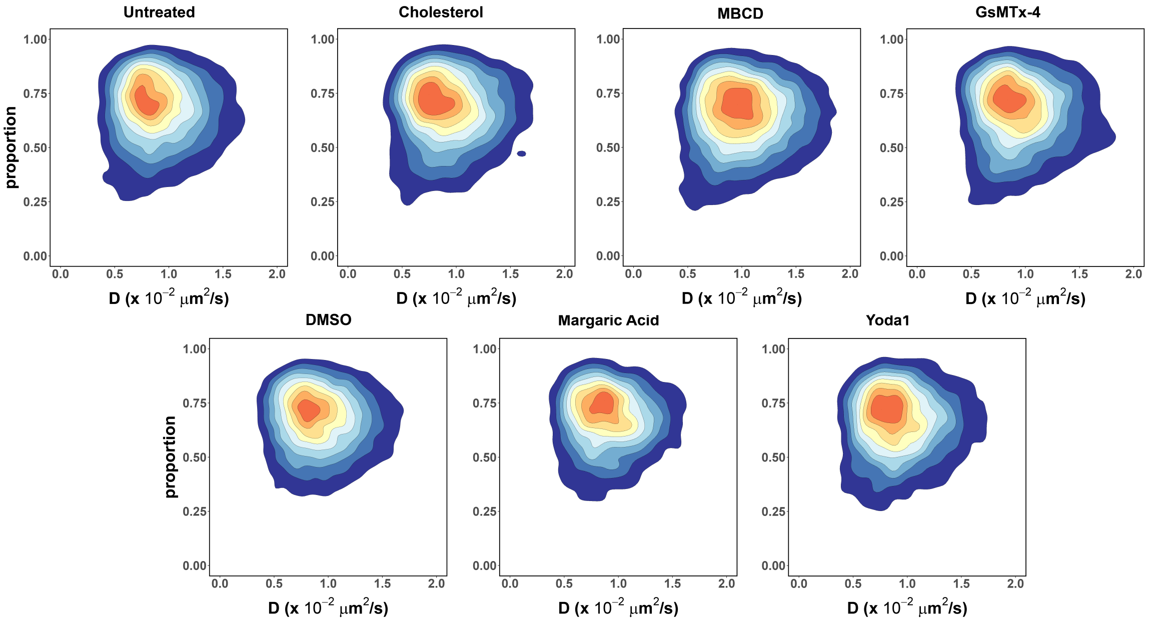

Supplemental Figure 3: The heterogeneity of PIEZO1-tdTomato immobile trajectories of the “slow” component remains largely unaltered upon treatment. The overall shape of the joint distribution of the parameters remains unchanged compared to the corresponding controls.

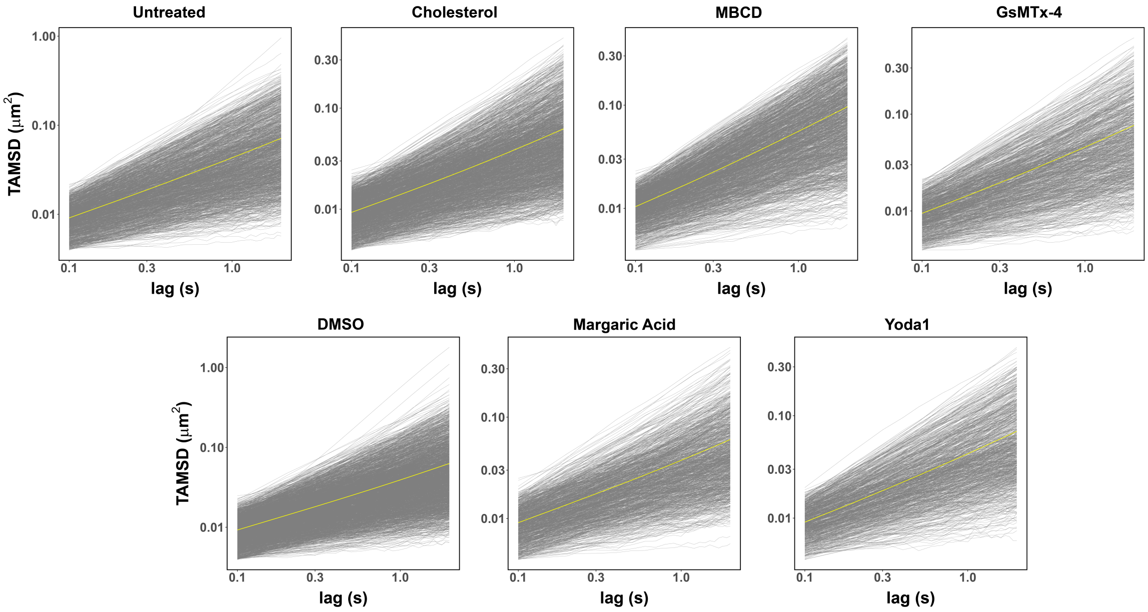

Supplemental Figure 4: The TAMSD as a function of time for single mobile trajectories of PIEZO1-tdTomato expressed in MEFs and treated with assorted drugs (a 1% sample of individual trajectories are shown in *gray* and the ensemble average TAMSD is shown in *yellow*).

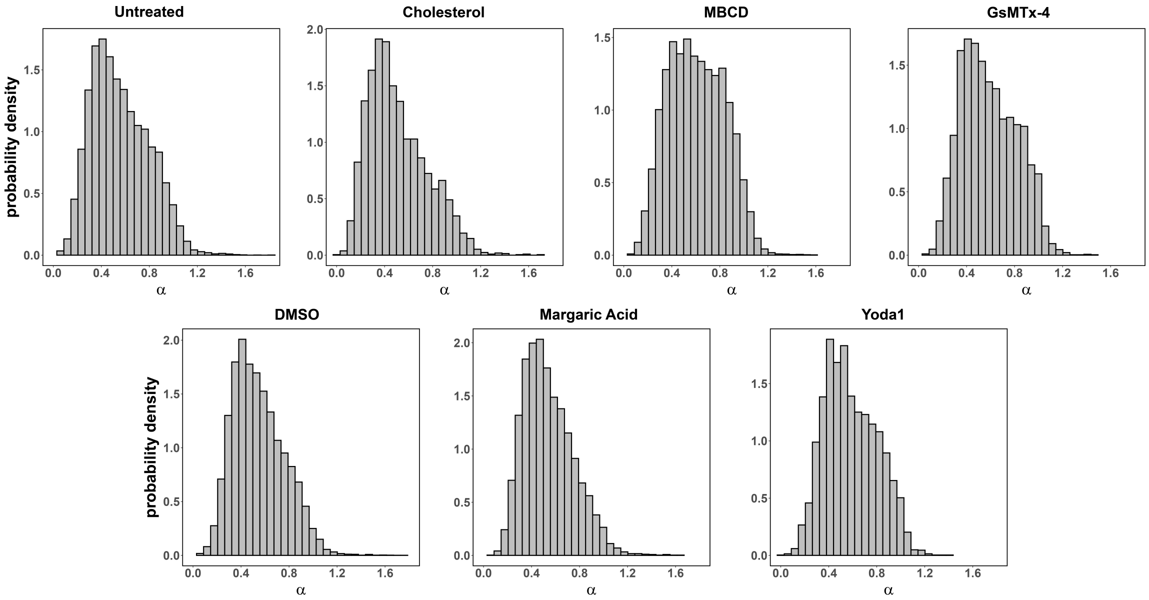

Supplemental Figure 5: The power-law exponents (α) describing single mobile trajectories TAMSD of PIEZO1-tdTomato expressed in MEFs treated with cholesterol, MBCD, GsMTx-4, margaric acid, Yoda1, and their respective controls are broadly distributed.

Supplemental Table 1: Contingency table used to calculate the mobile class odds ratio for drugs that can be compared with untreated control MEFs. All conditions were obtained over 3 experiments.

	Mobile	Immobile
MBCD	11097	5666
MBCD control	5906	3670
Cholesterol	3581	2745
Cholesterol control	4568	2867
GsMTx-4	5048	7506
GsMTx-4 control	1333	1281

Supplemental Table 2: Contingency table used to calculate the mobile class odds ratio for drugs that can be compared with DMSO-treated MEFs. All conditions were obtained over 3 experiments.

	Mobile	Immobile
Yoda1	4277	2058
Yoda1 control	3908	2427
Margaric acid	5982	3496
Margaric acid control	5846	3632

SUPPLEMENTARY VIDEOS

Supplementary Video 1: TIRF microscopy reveals heterogeneity in PIEZO1-tdTomato mobility. A. Representative TIRF image of PIEZO1-tdTomato puncta in live MEFs harvested from PIEZO1-tdTomato reporter mice. The white line denotes the cell boundary. Insets show enlarged regions of interest. The green inset is representative of regions where puncta appear mobile, whereas the blue inset is representative of regions where puncta show little or no mobility.
Scale bars = 10 µm.

Supplementary Video 2: TIRF microscopy videos of PIEZO1-tdTomato puncta from zoomed-in regions of live (left) and paraformaldehyde-fixed (right) MEFs. Trajectories are overlaid on to the video. Note the greater mobility of puncta in the live cell compared to fixed cell.
Scale bar = 1 µm.

SUPPLEMENTAL METHODS: VALIDATION OF TRAJECTORY GENERATION PARAMETERS

In order to guide and validate our trajectory generation parameters, we conducted several additional analyses, which we have detailed in this Supplemental Methods section. Prior to any analysis, we needed to select the ideal pixel linking distance between puncta. When linking trajectories, ID switching, a process in which puncta from different trajectories can be incorrectly linked, can occur when the pixel linking distance between puncta is too large. In this case, multiple trajectories could be combined into a larger trajectory, resulting in inaccurate trajectory measurements. On the other hand, trajectory fragmentation can occur when the pixel linking distance is too small. In this situation, trajectories generated will be incomplete and truncated. Thus, generating trajectories in Single Particle Tracking (SPT) studies involves an inherent trade-off between these two situations. These technical tests were performed on a small subset of our data (three representative experimental recordings, which we will now refer to as ”test data” in this section) in order to guide the parameters used to generate trajectories.

Based on visual inspection of the data, we first estimated that a three-pixel linking distance would be reasonable for trajectory generation. To test this choice, we measured the nearest neighbor (NN) distance between each linked particle and other particles in the image frame. The KDTree function from the scikit-learn library (75) was used to efficiently search for the NN to each punctum in a trajectory and the Euclidean distance was recorded (in pixels). This process was repeated for all frames in a single recording and the data summarized by the respective mean of each trajectory class (mobile or immobile). The resulting mean NN-distance values averaged over three separate recordings are shown in Supp. Methods Fig. 1A. In both trajectory classes, the mean NN-distance value is larger than the three-pixel distance used for linkage.

To quantify the displacement of linked puncta between consecutive frames, we computed the mean step length for each trajectory, given for an N-point trajectory, $\vec{x}(t) = \{\vec{x}_1, \vec{x}_2, \ldots, \vec{x}_N\}$, by $\sum_{i=1}^{N-1} |\vec{x}_{i+1} - \vec{x}_i|$. The calculations were performed using Flika (43) and a custom Python script. Similar to the NN distance calculation, we summarized the data from each recording by taking the means over each trajectory class. The resulting average values obtained from our test data are shown in shown in Supp. Methods Fig. 1B. In both trajectory classes, the mean step length is shorter than the three-pixel distance used for linkage, suggesting that we were able to successfully link most of the trajectories properly with a three-pixel linking distance.

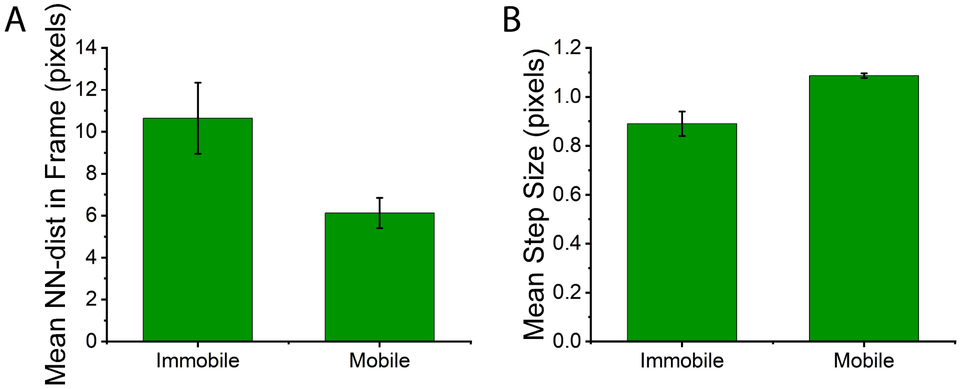

Supplemental Methods Figure 1: (A) Mean distance to the nearest-neighbor (NN) particle in a recording frame for each punctum in a trajectory. The mean NN distance between puncta in a recording frame for both immobile and mobile trajectories is larger than the three-pixel linking distance. (B) Mean step length between linked puncta in a trajectory. Trajectories from both (A) and (B) were generated using Flika and classified as mobile or immobile based on their sR_g values as described in Materials and Methods. The average of mean values from three 20 s recordings are shown, and the error bars are standard deviations.

We considered next the effect of the linkage distance values on trajectory classification. Using two second recordings from our test data, we implemented a range of pixel linking (1-20 pixels), which were generated using Flika (43) and a custom python script for iterating through the different parameters (Supp. Methods Fig. 2). Classification was performed as described in the manuscript. The two second recordings were truncated from the full-length recordings used in the manuscript to reduce the amount of computation required, which otherwise would have been restrictive.

We observed an increase in the proportion of mobile trajectories from $\tilde{2}5\%$ at a one-pixel linking distance to $\tilde{8}5\%$ at fifteen pixels and above, suggesting false-linkages between trajectories at higher pixel linkages and resulting in a classification of every trajectory as mobile. We similarly noted that the opposite effect occurred for immobile trajectories, hinting that these trajectories were falsely truncated at a one-pixel threshold. Thus, the three-pixel threshold (dashed blue line in Supp. Methods Fig. 2) provided a good balance for capturing correct vs false links.

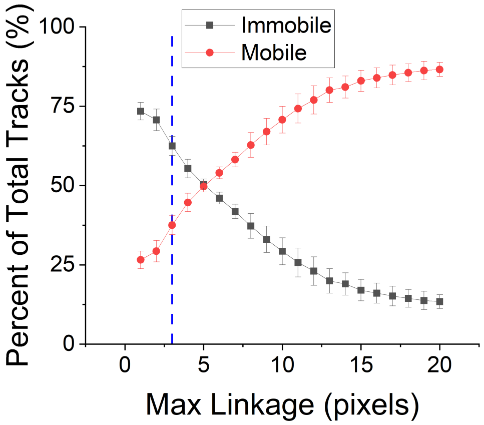

Supplemental Methods Figure 2: The effect of linkage distance value on trajectory classification. Trajectories were established for 2 s test data using a range of linkage parameters (1-20 pixels). The plot shows mean values over the three recordings. The error bars are standard deviations. The dashed blue line is indicative of 3-pixel linkage, which we use in our analyses.

While a three-pixel cutoff may result in some truncated trajectories and an underestimation of actual trajectory mobility, this does not affect the main findings of the paper as the majority of step sizes fall below three pixels in the mobile class. Together, these analyses demonstrate that the three-pixel threshold provides a good balance between ID switching and fragmented trajectories.

CODE AVAILABILITY

The code used for the trajectory analysis is available on GitHub at
https://github.com/Pathak-Lab/Piezo1-tdTomato-Trajectory-Analysis
